# Soil biome variation of *Lupinus nipomensis* in wet‐cool vs. dry‐warm microhabitats and greenhouse

**DOI:** 10.1002/ajb2.70020

**Published:** 2025-03-21

**Authors:** Peter T. Nguyen, Justin C. Luong, Van Wishingrad, Lisa Stratton, Michael E. Loik, Rachel S. Meyer

**Affiliations:** ^1^ Department of Ecology and Evolutionary Biology University of California Santa Cruz Santa Cruz 95064 CA USA; ^2^ Department of Life and Environmental Sciences University of California Merced Merced 95340 CA USA; ^3^ Department of Forestry, Fire & Rangeland Management California State Polytechnic University, Humboldt Arcata 95521 CA USA; ^4^ Department of Environmental Studies University of California Santa Cruz Santa Cruz 95064 CA USA; ^5^ Vernon and Mary Cheadle Center for Biodiversity and Ecological Restoration University of California Santa Barbara 93105 CA USA; ^6^ Hawai'i Institute of Marine Biology 46‐007 Lilipuna Road, Kaneohe Hawai'i 96744 USA

**Keywords:** drought tolerance, ecological reserve, endangered species, Fabaceae, greenhouse, metabarcoding, microclimate, plant conservation, plant‐microbe interactions

## Abstract

**Premise:**

Environmental DNA (eDNA) can be used to determine the composition of the soil biome community, revealing beneficial and antagonistic microbes and invertebrates associated with plants. eDNA analyses can complement traditional soil community studies, offering more comprehensive information for conservation practitioners. Studies are also needed to examine differences between field and greenhouse soil biomes because greenhouse‐grown plants are often transplanted in the field during restoration efforts.

**Methods:**

We used eDNA multilocus metabarcoding to test how the soil biome of the federally and state‐endangered species, *Lupinus nipomensis*, differed between wet‐cool and dry‐warm microhabitats. At Arroyo Grande, California, 20 experimental plots were sampled, representing a factorial combination of wet‐cool vs. dry‐warm soil and plots that did or did not contain *L. nipomensis*. In a simultaneous greenhouse study, *L. nipomensis* was grown in drought and well‐watered conditions to compare soil communities between field and greenhouse.

**Results:**

A diversity of carbon‐cycling microorganisms but not nitrogen‐fixers were overrepresented in the field, and nitrogen‐fixing bacteria were overrepresented in some greenhouse treatments. The microbial communities in the field soils were more species‐rich and evenly distributed than in greenhouse communities. In field plots, microhabitats significantly influenced community beta diversity, while field plots with or without *L. nipomensis* had no significant differences in alpha or beta diversity.

**Conclusions:**

Our study shows the utility of eDNA soil analysis in elucidating soil biome community differences for conservation and highlights the influence of plant microhabitats on soil microbe associations.

The soil biome, encompassing invertebrate and microbial communities such as bacteria, fungi, archaea, invertebrates and protists, plays a crucial role in supporting plant growth by regulating nutrient fluxes, water‐use efficiency, and drought resistance (Fierer, 2017; Bastida et al., 2020). The soil biome interacts with physiological and morphological changes in plant traits, helps regulate essential nutrients for plant growth, and interacts with climate stressors (Sayer et al., 2017; Jansson and Hofmockel, 2020). These interactions become particularly important during periods of climate stress, where the soil microbiome can influence plant success and conservation outcomes (Dubey et al., 2019; Liu et al., 2020), not only through influencing growth and abiotic stress tolerance, but in regulating disease and reproduction as well (Waldrop and Firestone, 2006). Furthermore, microbiome enzyme activity and biomass in the rhizosphere can be reduced by drought and heatwaves (Waldrop and Firestone, 2006), creating a feedback loop on soil patterns and processes.

The increasing frequency of droughts and heatwaves is impacting ecosystems globally, not only by potentially reducing plant growth but also by complicating efforts to reintroduce rare plants, especially in semiarid regions such as California (Hulley et al., 2020). Climate stressors such as drought and heat waves disrupt water availability and nutrient cycles, reducing biodiversity (Pascual et al., 2022). Furthermore, increases in soil temperature can induce water‐related stresses and hinder the growth of plants. In such climates, leaves may exhibit lower nutrient resorption during periods of warming and reduced rainfall (Prieto and Querejeta, 2020), impacting plant morphology and physiological traits such as leaf gas exchange (Anjum et al., 2011; Luong and Loik, 2022). Therefore, endangered plants in California are particularly vulnerable to these climate‐induced changes (Bartholomeus et al., 2011). The specific challenges faced by these plants suggest that plants need to establish relationships with other organisms to mitigate climate stress (Chamard et al., 2024). Microbiome studies may help explain why drought exacerbates plant mortality in low‐nitrogen environments (Zhang et al., 2007; Abid et al., 2016).

The microbiome for legumes is seldom studied outside of agricultural species but are particularly intriguing because of their unique nitrogen‐fixing abilities (Mahmud et al., 2020). For example, the legume *Pisum sativum* L. (Fabaceae) is more productive and drought resilient in agricultural environments with high bacterial and fungal diversity (Prudent et al., 2020). Some microbiome studies have shown that certain organisms are associated with drought tolerance (Busby et al., 2017). For example, *Triticum aestivum* L. (wheat; Poaceae) has a beneficial relationship with soil bacteria that increases drought tolerance (Hone et al., 2021). Additionally, soil invertebrates like earthworms can both mitigate and exacerbate the effects of abiotic stressors such as drought, impacting both plant and insect communities (Johnson et al., 2011). Coupled with environmental effects, legacy effects play a large role in soil biome community composition. Soil microbial legacies significantly influence future plant growth, either boosting or hindering development based on their nature and interactions (Hannula et al., 2021). Overall, traditional microbiome–drought–plant associations and soil inoculum trials can benefit from eDNA sampling for a comprehensive view of in‐field soil biome associations (Yang et al., 2024).

In contrast to field studies, greenhouse experiments have historically been used to better understand how plants respond to various climate stresses in a controlled environment (Gibson et al., 1999). However, researchers have not found a strong correlation between greenhouse‐ and field‐measured plant–soil feedbacks (Forero et al., 2019), which raises a concern for endangered plant reintroduction efforts, which typically involve *ex situ* cultivation using soil from sources outside the reintroduction site, overlooking the significance of the soil biome. Given the importance of microbial communities in nutrient cycling and plant growth (Jansson and Hofmockel, 2020) and soil invertebrate communities in recycling organic matter (McGee et al., 2020), studies are needed to understand the role of plant–soil‐biome relationships for in situ and ex situ conservation efforts. A recent molecular microbiome analysis of the soil from rare legumes in another semiarid location suggests that microbial effects are predictive of germination and survival rates (Hodgson et al., 2023). This emerging knowledge shows the utility of molecular tools for uncovering intricate interactions within soil biomes.

Building on these insights, molecular methods can improve our understanding of plant soil‐biome communities and inform conservation efforts during intensifying climate change (Trevelline et al., 2019). Even though the soil biome may play a crucial role in the life history of plants, they are often challenging to study with traditional field methods (Berg et al., 2020). Some commonly measured belowground traits such as specific root length have limited functional relevance compared to complex mycorrhizal associations, and thus researchers may overlook vital drivers of plant and ecosystem functioning (Trevelline et al., 2019; Freschet et al., 2021). In fields such as forensic ecology, genetics, and metagenomics, environmental DNA (eDNA) is a tool to profile biodiversity and identify organisms in samples from soil, air, and water (Rees et al., 2014; Johnson et al., 2023). eDNA metabarcoding is used to inventory microbes and invertebrates, which complements visual and culturable surveys to identify which taxa play key roles in assisting plants in resource allocation (Rees et al., 2014; Deiner et al., 2017).

We used eDNA to study the microbial and invertebrate communities in bulk soil (adjacent to the roots) of the endangered legume *Lupinus nipomensis* Eastw. (Fabaceae; common name Nipomo Mesa lupine). This species is listed as endangered by the United States (3/20/2000, USFWS 65 FR 14888 14898) and endangered by the state of California (CNDDB record PDFAB2B550). California's rising temperatures, invasion of exotic plants such as *Ehrharta calycina*, and the limited habitat range of *L. nipomensis* make it an ideal candidate for a study of the soil microbiome. We asked: (1) At our study site, do soil biome communities differ in composition between microhabitats that are relatively wet and cool compared to those that are dry and warm? (2) Are there differences in soil biome community composition between plots that contain and those that do not contain *L. nipomensis*? (3) Are there differences in soil‐biome community profiles between greenhouse soils and field‐collected soils? We hypothesized that relatively wet‐cooler microhabitats and plots that contain *L. nipomensis* would have a significantly larger and more diverse soil biome because the greater moisture and cooler temperatures in these microhabitats are likely to enhance nutrient availability and microbial activity. We predicted that field‐soil biome communities would have more alpha diversity (richness and evenness) and beta diversity (composition) compared to those in greenhouse soils, with little to no overlap in community composition.

## MATERIALS AND METHODS

### Study species

*Lupinus nipomensis* (Fabaceae) is an annual legume endemic to the Guadalupe‐Nipomo Dunes of California, United States. It is located in southern coastal San Luis Obispo County, within the Guadalupe‐Nipomo Dune Complex (35.055° N, –120.604° W, 13 m a.s.l.), where it occupies back dune and interdune habitats. This species is currently found in only a single population, consisting of three occurrences near Nipomo, CA with one extant and the other two the result of outplanting efforts for its conservation. Loss of coastal back dune habitat and climate stressors had caused the *L. nipomensis* population to decline (Skinner and Pavlik, 1994). This study was permitted under United States Fish and Wildlife Service permit 2081(a)‐21‐023‐RP issued to Luong, Stratton, and Nguyen.

### Field study and soil sampling

We conducted the field portion of our study at Black Lake Ecological Area (BLEA) in Arroyo Grande, California (35.054972° N, 120.603647° W, 25 m a.s.l.). BLEA is owned and managed by The Land Conservancy of San Luis Obispo and occurs within the Guadalupe‐Nipomo Dune Complex. It is situated 30 km inland from the coast and has a mediterranean climate that experiences dry‐warm summers and wet‐cool winters (Appendix S1). The predominant soil type within the dunes is a fine sandy loam, and the dunes are characterized by the absence of hydric soils. The invasive veldt grass *Ehrharta calycina* dominates the local dune systems. In the summer months of June through August, the area receives 4 mm of total rainfall and averages 15.9°C, and in the winter (December, January, February), the area receives 231 mm of rainfall and averages 12.1°C (PRISM Climate Group, 2014). The location on the coast means low‐lying stratocumulus clouds and fog provide shade, humidity, and cooler temperatures for plants in the dry‐warm summers (Rastogi et al., 2016). Experimental plots were established in 2014 to assess how microhabitats influence the reintroduction of *L. nipomensis* (Luong et al., 2019).

We selected a subset of 20 experimental plots to sample eDNA from a seeding trial established in 2014 with caging treatments on different microhabitat conditions (see Luong et al., 2019). Plots were 10–20 m apart, and each represented one of four conditions (*N* = 5 plots per condition): wet‐cool containing *L. nipomensis*, wet‐cool not containing *L. nipomensis*, dry‐warm containing *L. nipomensis*, and dry‐warm not containing *L. nipomensis* (Luong and Stratton, 2016; Stratton et al., 2022). After the first outplanting efforts, 24 plots contained *L. nipomensis*, while the other plots no longer had *L. nipomensis* present. We used survival and reproduction data from Luong et al. (2019) and Stratton et al. (2022) to locate and identify plots where *L. nipomensis* was present (+Ln) and plots where *L. nipomensis* was not (‐Ln) (Appendix S2). Wet‐cool areas are found on north‐facing slopes and swales, while warm‐dry areas are on south‐facing slopes and dune ridges (Braatne and Bliss, 1999; Bennie et al., 2006). Using this information, we identified two microhabitats categorized by a combination of slope and aspect: cool‐wet and warm‐dry to compare community composition differences. In 2020 from January to April (growth to flowering stages), soil temperature and volumetric water content (VWC) data were collected and showed that wet‐cool microhabitats were on average 11°C, while our dry‐warm microhabitats were 15°C. This temperature difference is comparable to the ~4°C temperature experimental manipulation in our greenhouse warming treatment (next section). Furthermore, our wet‐cool sites had an average volumetric water content (VWC) of 4.6%, which was higher than the average VWC of 1.8% in the dry‐warm sites (*F*_2,39_ = 32.84, *P* < 0.001). Sandy soils tend to hold less moisture, which results in smaller shifts for VWC and temperature and could more strongly affect soil biome communities (Al‐Kaisi et al., 2017).

At each of the 20 plots selected for soil eDNA sampling, we used three 2‐mL cryotubes to collect the soil samples (*N* = 60), where three tubes represent one sample. Sampling was done on 10 and 23 March 2022. We switched gloves after each collection to avoid cross‐contamination. The samples were taken ~5 cm apart at a depth of ~5 cm, with effort made to avoid any plant roots. The samples were then placed on ice and transferred to a –19°C freezer where they were stored until DNA extraction.

### Greenhouse study and soil sampling

In the University of California, Santa Cruz Jean H. Langenheim Greenhouses, we grew eight *L. nipomensis* individuals in a 1:1 mix of Pro‐Mix HP Agtiv Reach (Premier Tech Horticulture, Rivière‐du‐Loup, Canada) and high porosity, sterile sand in 107 mL Ray Leach Stubby Cell Classic containers (0.21 × 0.03 m; Stuewe & Sons, Tangent, OR, USA). Plants were grown from December 2021 to April 2022 with an average temperature of 21°C during the day and 18.7°C at night in early winter (December–February) and 20.2°C during the day and 13.1°C at night in early spring (March–April) at a relative humidity of ~70%. No supplemental lighting was used, plant positions were randomized weekly to minimize position effects, and individuals were irrigated and fertilized with 2.5 mL 20‐0‐0 fertilizer (Premier Tech Horticulture, Rivière‐du‐Loup, Canada) per 3.78 L water weekly until we started the treatments. This study used a factorial design with two treatment variables: warming (+4°C) and episodic drought. The warming treatment was applied by placing plants under a polycarbonate cube paired with a heat lamp, with cone‐tainers randomized weekly to account for microclimate variations because no supplemental lighting was used. The episodic drought treatment was imposed by withholding water until plants reached a threshold stomatal conductance of 0.05 mol H₂O m⁻² s⁻¹ (Duan et al., 2014; Luong and Loik, 2022), then they were re‐watered. We collected three 2‐mL cryotubes of surface soil as a single sample per pot after ~160 days of treatment. Soil samples were collected 3 cm from the base of the plant at a depth of 5 cm.

### DNA extraction, amplification, and sequencing

We pooled ~0.5 g of soil from each of the three 2‐mL replicate cryotubes for each sample for an average representation of the microbial community composition at each collection site or pot before the DNA extraction. Pools were homogenized by vortexing, and 0.25 g was used in DNA extraction with the Qiagen DNeasy PowerSoil kit (Qiagen, Germantown, MD, USA) following the manufacturer's protocol. We used the CALeDNA methods (Meyer et al., 2021) to generate multilocus metabarcoding libraries, targeting the ITS1 region from fungi (hereafter, FITS) (White et al., 1990; Epp et al., 2012), *CO1* from invertebrates and protists (Yu et al., 2012; Leray et al., 2013), 18S rRNA from eukaryotes more broadly (Amaral‐Zettler et al., 2009), and 16S rRNA from bacteria and archaea (Caporaso et al., 2012) (Appendix S3). After DNA extraction, all lab work was done in a HEPA‐filtered PCR hood pre‐sterilized with DNA AWAY (Thermo Fisher Scientific, Waltham, MA, USA), 20% v/v bleach, and 70% v/v ethanol, then UV light for 20 min. New nitrile gloves and vinyl‐sterilized sleeves were worn at all times to minimize contamination. All primers had Nextera adapters (Illumina, San Diego, CA, USA) on the 5′ end. The PCRs were done in triplicate per metabarcode in 15‐µL reaction volumes with 2 μL of DNA template with 0.15 μL of each 10 μM primer and 7.5 μL Qiagen Multiplex PCR Master Mix polymerase. The PCRs for 16S and 18S rRNAs and ITS were run in a ProFlex PCR Systems Thermocycler (Applied Biosystems, Thermo Fisher Scientific, Waltham, MA, USA) used the following conditions: initial activation at 95°C for 5 min; touchdown phase of 13 cycles of 94°C for 30 s, annealing from 69.5°C decreasing by 1.5°C per cycle for 30 s, extension at 72°C for 60 s; 35 cycles at 94°C for 30 s, 50°C for 30 s, 72°C for 60 s; final extension at 72°C for 10 min, followed by a hold at 10°C indefinitely. The PCR for *CO1* included an initial activation at 95°C for 5 min; touchdown phase of 13 cycles of annealing from 69.5°C decreasing by 1.5°C per cycle for 90 s, extension at 72°C for 90 s; amplification phase of 40 cycles. Other steps and temperatures were the same as the previous protocol. PCR replicates were pooled and cleaned with MagBio HighPrep PCR beads (MagBio Genomics, Gaithersburg, MD, USA). A Qubit Broad Range assay (Thermo Fisher) was used to quantify the cleaned product. Equimolar quantities of the four marker amplicons were pooled by sample. We ran indexing PCRs in 25‐μL volumes that contained 1.25 μL of Illumina Nextera unique dual indexes (Illumina) and 12.5 μL of Kapa HiFi HotStart Ready Mix (Roche, Indianapolis, IN, USA) and between 10 and 100 ng pooled PCR product. The PCR was run for 5 min for an initial cycle of denaturation at 95°C, 12 cycles of denaturation for 20 s at 98°C, annealing for 30 s at 56°C, and extension for 3 min at 72°C, with a final round of extension at 72°C for 5 min, and subsequently held at 8°C until removal. Libraries were analyzed by gel electrophoresis, size‐selected once more with beads to remove <200‐bp fragments, and quantified using a Qubit Broad Range assay. Libraries were pooled to have the same number of molecules and sequenced on an Illumina MiSeq with a target depth of 50,000 reads per sample per metabarcode.

### Data analysis

We sequenced 20 samples from BLEA in the field, eight greenhouse samples, two DNA extraction blanks, and a PCR blank. We used the Anacapa Toolkit (Curd et al., 2019) to quantify and sort reads, assign amplicon sequence variants (ASVs), and assign taxa to the samples using the Crux version 2 reference databases (https://ucedna.com/reference-databases-for-metabarcoding). Reference databases were created in October 2022 using CRUX in the Anacapa Toolkit (Curd et al., 2019). The Anacapa Toolkit employs CutAdapt (Martin, 2011) and the FastX‐Toolkit (A. Gordon, Cold Spring Harbor Laboratory, 2010, unpublished software) for initial Fastq quality control and filtering, then assigns ASVs and detects and removes chimeras using DADA2 (Callahan et al., 2016). For the taxonomic identification of each ASV, Anacapa uses Bowtie2 (Langmead and Salzberg, 2012) alongside the Bayesian lowest common ancestor (BLCA) algorithm (Gao et al., 2017), which we set to a Bayesian confidence cutoff of 80. We prepared phyloseq objects for analysis using R (Version 4.2.2, R Core Team, 2022) and R packages ranacapa (Kandlikar et al., 2018) and phyloseq (McMurdie and Holmes, 2013). We used the R package decontam (Davis et al., 2018) to remove contaminant reads and taxa from the taxonomy tables using the prevalence method and a threshold = 0.1 to exclude sequencing reads that are most likely from contamination during PCR and extractions. Decontamination removed seven taxa from 16S, two taxa from 18S, 0 from *CO1*, and two from FITS. Then, we only retained taxa found in at least two samples and with at least 20 reads in the total data set for the metabarcode. Decontaminated data were plotted as rarefaction curves, and a minimum depth of 1000 reads was chosen as a criterion to retain samples, rather than using rarefaction because 1000 reads included most samples reaching the log phase of the curve. With minimum‐depth filtered phyloseq objects, the R package vegan (Dixon, 2003; Oksanen et al., 2022) was used to generate diversity indices and the ggplot2 package (Wickham, 2011) was used to visualize the results.

We calculated Shannon and Chao1 indices to measure taxonomic alpha diversity, using the phyloseq estimate_richness function. While the Shannon estimator considers species evenness, Chao1 considers singletons (unique or rare species or taxa that are observed only once or twice within a given data set) and the number of observed species. We used the R package stats (R Core Team, 2022) to assess the distributional assumptions of our data with a Shapiro–Wilk test. After confirming that the parametric assumptions were met, we conducted two‐tailed, independent *t*‐tests to assess the potential relationships between the Shannon and Chao1 diversity measurements and the variables of interest.

We used MicrobiomeSeq (Ssekagiri et al., 2017) to plot relative abundance bar plots and to measure the relative contribution of each sample to beta diversity. We used these techniques to study the quantity, distribution, and changes in several microbial taxa within a community and understand their composition and differences between samples through plots and measurements. We calculated differential abundance of specific taxa across pairs of sample groups with DESeq2 (Love et al., 2014) and compared results to relative abundance plots. DESeq2 can be used to determine which microbial taxa are overrepresented, meaning they are more or less prevalent in certain samples or groups. When a taxon is overrepresented, it signifies that there are more of that taxon in that treatment (i.e., wet‐cool microhabitat) than would be predicted by chance alone, showing that the treatment is likely affecting that taxon's expression. DESeq2 was run with settings fittype = parametric, test = Wald, and alpha = 0.15. A positive log_2_ FoldChange indicates that microbial taxa from a wet‐cool microhabitat are overrepresented, while a negative value indicates taxa from the dry‐warm microhabitat are overrepresented.

We used the betadiver function in the R package vegan (Oksanen et al., 2022) to calculate Jaccard dissimilarity indices and to analyze the taxonomic diversity and composition of the communities for each metabarcode. The Jaccard distance is a metric for analyzing taxonomic composition and uses presence/absence data, which describes community turnover insensitive to abundance variations (Zhou and Ning, 2017). We used principal coordinate analysis of Jaccard dissimilarities to examine the composition and turnover of the microbial community, to visualize the patterns of similarity and dissimilarity within the data, and to identify trends in community composition. We employed a PERMANOVA to assess how dissimilarities were related to microhabitats and the presence of *L. nipomensis*. We used the betadisper function in vegan with 99 permutations in the permutest to look for differences in beta dispersion (Oksanen et al., 2022). Beta dispersion is a statistical measure to assess variation in taxonomic composition across different sites, habitats, or time points, revealing ecological differentiation and turnover (Anderson et al., 2006). It evaluates the impact of environmental factors or disturbances on community structure by quantifying the dissimilarity between sampling units within groups. Bonferroni adjustments were applied to the *P*‐values.

## RESULTS

### Metabarcoding result summaries

From the 28 sample libraries and three negative controls, we obtained 7.4 million reads. After quality control, taxonomy assignment, decontamination, taxon filtering, and minimum read‐depth sample filtering, we retained 2 million assigned reads. These were parsed as 555 taxa and 276,605 reads for 16S (bacteria and archaea), 377 taxa and 485,380 reads for 18S (broad eukaryotes), 94 taxa and 716,947 reads for *CO1* (largely protists and invertebrates), and 273 taxa and 503,840 reads for FITS (fungi). Taxonomic results are reported in Appendix S4, and all ASVs and their assignments are included in the Zenodo depository (https://doi.org/10.5281/zenodo.12216630). In our results, the 16S marker showed 491 taxonomic lineages (i.e., most resolved taxa) in the greenhouse and 520 from the field population, with 456 shared between the two. The 18S results showed 186 taxonomic lineages in greenhouses, 333 from the field, and 143 shared. The *CO1* results showed 42 taxonomic lineages in greenhouses, 84 from the field, and 33 shared. FITS showed 72 taxonomic lineages in the greenhouse, 239 from the field site, and 42 shared.

### Field soil eDNA composition differs between microhabitats

**Figure 1:**
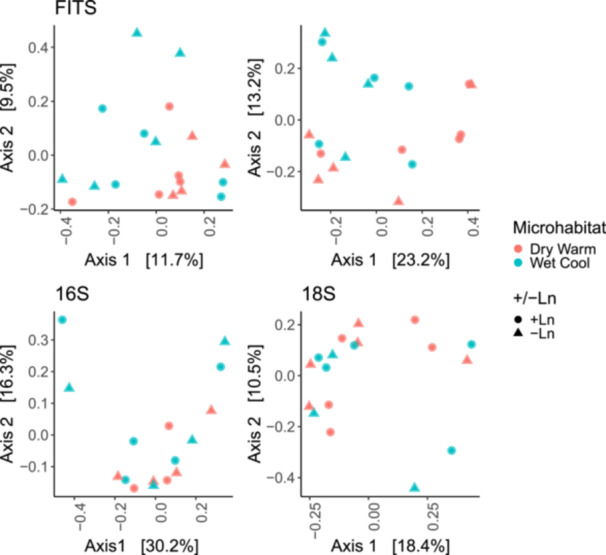
The PCoA for fungal ITS, 16S and 18S rRNAs, and *CO1* shows the relationship between samples based on their taxonomic composition in Black Lake Ecological Area plots of the taxonomic beta diversity. The PCoA plot indicates a separation between samples based on microhabitat, especially evident in *CO1* along PCo2 and FITS along PCo1 where microhabitat samples tend to group with each other, and Wet Cool samples have more dissimilarity (which we follow up with in beta dispersion tests later). The PCoA uses transformed *x‐* and *y*‐axes to represent the coordinates of samples in a multidimensional space, while the accompanying percentages signify the relative amount of variation accounted for by each axis. +Ln: plots with *Lupinus nipomensis*; −Ln: plots with no *L. nipomensis*.

**Figure 2:**
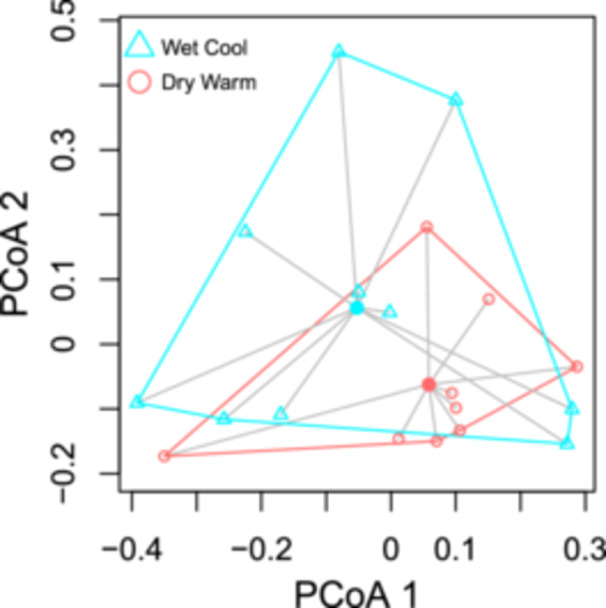
Beta dispersion of 16S rRNA at Black Lake Ecological Area when comparing microhabitats. Dry‐warm microhabitats were significantly smaller and condensed when compared to wet‐cool microhabitats (*P* = 0.02). Distance between the points reflects the dissimilarity in species composition between samples. The polygons on the plot represent community clusters with similar species composition, meaning the larger the polygon the larger the variation.

Our comparison of wet‐cool and dry‐warm microhabitat community composition (Figure 1) revealed differences in taxonomic beta diversity in the *CO1* PERMANOVA results (Table 1), but no differences in beta dispersion (Appendix S5). FITS results showed marginally significant differences between wet‐cool and dry‐warm microhabitats (Table 1), but no differences in beta dispersion (Appendix S5). Beta diversity did not differ for 16S and 18S between microhabitats, but 16S did exhibit less beta dispersion in the warm‐dry group compared to the wet‐cool group (Figure 2; Appendix S5). We found no differences in taxonomic beta diversity or beta dispersion for *CO1*, 18S, FITS, or 16S between +Ln and −Ln plots in the field (Table 1; Appendix S5). Examining alpha diversity indices, we found no differences in the Chao1 or Shannon metrics in any markers when comparing microhabitats or between +Ln and −Ln (Table 2).

**Table 1:** PERMANOVA results for beta diversity using the Jaccard dissimilarity index for 16S and 18S rRNAs, fungal ITS (FITS), and *CO1*. The microhabitat variable tests wet‐cool versus dry‐warm microhabitats. The +/−Ln variable tests +Ln (with *Lupinus nipomensis*) and −Ln (without *L. nipomensis*) plots. FITS and *CO1* were significant when testing for microhabitat.

Variable	Marker	df	*F*	*P*
Microhabitat	16S	17.000	0.958	0.512
Microhabitat	18S	17.000	0.958	0.500
Microhabitat	FITS	15.000	1.253	0.055
Microhabitat	*CO1*	15.000	1.751	0.037
+/−Ln	16S	17.000	0.772	0.910
+/−Ln	18S	17.000	0.772	0.907
+/−Ln	FITS	15.000	0.913	0.684
+/−Ln	*CO1*	15.000	1.267	0.173

**Table 2:** Results of *t*‐test for alpha diversity using the Shannon index and Chao1 estimator for 16S and 18S rRNAs, fungal ITS (FITS), and *CO1*. The microhabitat variable tests wet‐cool versus dry‐warm microhabitats. The +/−Ln variable tests +Ln (with *Lupinus nipomensis*) and −Ln (without *L. nipomensis*) plots. There were no significant interactions.

Index	Variable	Marker	df	*t*	*P*
Chao1	Microhabitat	16S	10.370	0.025	0.981
Chao1	Microhabitat	18S	14.660	–0.341	0.749
Chao1	Microhabitat	FITS	16.930	0.921	0.369
Chao1	Microhabitat	*CO1*	16.970	–0.127	0.900
Chao1	+/−Ln	16S	13.710	0.451	0.659
Chao1	+/−Ln	18S	14.660	–0.326	0.749
Chao1	+/−Ln	FITS	16.020	0.241	0.813
Chao1	+/−Ln	*CO1*	16.720	–0.170	0.867
Shannon	Microhabitat	16S	10.910	–0.189	0.854
Shannon	Microhabitat	18S	15.150	–0.083	0.935
Shannon	Microhabitat	FITS	14.140	1.531	0.147
Shannon	Microhabitat	*CO1*	16.930	–0.281	0.782
Shannon	+/−Ln	16S	9.550	1.549	0.154
Shannon	+/−Ln	18S	9.220	0.421	0.683
Shannon	+/−Ln	FITS	16.940	0.479	0.638
Shannon	+/−Ln	*CO1*	16.970	–0.027	0.978

### BLEA eDNA taxa are differentially represented in microhabitats and + Ln/−Ln test groups

In the field, DESeq2 analysis revealed that five taxa were overrepresented in wet‐cool plot samples (Table 3): two bacteria that could not be resolved to species, two fungal taxa identified using *CO1* sequences, and one parasitic protist from the Plasmodiophoridae identified using 18S sequences. Only two taxa were overrepresented in the dry‐warm plots: a bacterium in class Pseudonocardiaceae and an unknown fungus in Dothideomycetes. We identified one bacterial taxon in the family Geodermatophilaceae overrepresented in −Ln plots and one fungal taxon each in the family Glomerellaceae and Lasiosphaeriaceae overrepresented in +Ln plots (Table 3). Functional information for these taxa can be found in Appendix S5.

**Table 3:** Taxa identified using DESeq2 for 16S and 18S rRNAs, fungal ITS (FITS), and *CO1* and found to be over‐ or underrepresented across microhabitats and plots with *Lupinus nipomensis* (+Ln) and without (−Ln). A positive log_2_ FoldChange suggests microbes are overrepresented in the wet‐cool microhabitat; a negative log_2_ FoldChange suggests microbes are overrepresented in the dry‐warm microhabitats. The last column indicates mean read abundance.

Marker	Family	Lowest taxonomic rank	log_2_ FoldChange	*P*	Mean read abundance, +SD
**Overrepresented in wet‐cool (WC)**					
16S	Verrucomicrobia subdivision 3	—	1.33	0.110	WC 19.7, 16.92; DW 6.4, 6.19
16S	Unknown Gammaproteobacteria (class)	—	0.97	0.040	WC 72.7, 48.72; DW 31.1, 26.23
*CO1*	Aspergillaceae	*Penicillium sclerotiorum*	5.89	0.110	WC 223.3, 313.74; DW 7.8, 23.97
*CO1*	Erysiphaceae	*Blumeria graminis*	5.34	0.140	WC 53.3, 83.56; DW 18.2, 56.85
18S	Plasmodiophoridae	*Spongospora subterranea*	6.32	0.040	WC 322.1, 676.67; DW 1.7, 5.38
**Overrepresented in dry‐warm (DW)**					
16S	Pseudonocardiaceae	—	−2.04	0.040	WC 13.6, 8.6; DW 70, 80.51
FITS	Dothideomycetes (Class)	—	−3.51	0.140	WC 4.1, 4.28; DW 40.2, 40.04
**Overrepresented in ‐Ln plots**					
16S	Geodermatophilaceae	*Modestobacter* (genus)	−2.63	0.040	+Ln 49.3, 102.03; −Ln 79.4, 128.18
**Overrepresented in** + **Ln plots**					
FITS	Lasiosphaeriaceae	*Cladorrhinum australe*	23.45	<0.001	+Ln 21.3, 53.97; −Ln 9.5, 30.4
*CO1*	Glomerellaceae	*Colletotrichum salicis*	24.16	<0.001	+Ln 29.6, 77.08; −Ln 14.8, 46.8

### Comparison of greenhouse eDNA communities between well‐watered and drought treatments

Many taxa were overrepresented in our greenhouse study in the well‐watered and the drought treatments. In the well‐watered treatments, we found overrepresented taxa in the families Acidobacteriaceae (*P* = 0.040), Gemmatimonadaceae (*P* = 0.002), Helotiaceae (*P* < 0.001), Stachybotryaceae (*P* < 0.001), and Ascomycota (*P* = 0.011) and the orders Nitrosomonadales (*P* = 0.002) and Hypocreales (*P* = 0.003) (Table 4). In drought treatments, we found overrepresented taxa in families Burkholderiaceae (*P* = 0.048), Micropepsaceae (*P* < 0.001), and Oxalobacteraceae (*P* = 0.019). Notably, three taxa with known functions in decomposition, disease, and nutrient uptake were high in well‐watered treatments compared to other overrepresented taxa: Helotiaceae (log_2_ FoldChange = 22.5), and Stachybotryaceae (log_2_ FoldChange = 24.2) and order Hypocreales (log_2_ FoldChange = 9.62), respectively (Table 4; Appendix S6).

**Table 4:** Taxa identified using DESeq2 for 16S, 18S, fungal ITS (FITS), and *CO1* taxa that were over‐ or underrepresented across well‐watered and droughted greenhouse samples. A positive log_2_ FoldChange suggests microbes are overrepresented in the well‐watered soil samples, while a negative log_2_ FoldChange suggests microbes are overrepresented in the droughted soil samples. The last column indicates the most resolved classification based on alignments to our reference DNA databases.

Marker	Family	Lower classification	Log_2_ FoldChange	*P*	Mean read abundance, +SD
**Overrepresented in well‐watered (WW)**					
16S	Acidobacteriaceae	*bacterium Ellin6099*	0.98	0.040	WW 121.0, 43.49; D 35.0, 7.81
16S	Gemmatimonadaceae	*Gemmatimonas* (genus)	1.13	0.002	WW 175.25, 53.50; D 47.75,15.79
16S	Nitrosomonadales (order)	—	2.63	0.002	WW 78.5, 42.43; D 7.25, 4.82
*CO1*	Helotiaceae	*Articulospora tetracladia*	22.5	<0.001	WW 137.5, 238.13; D 0, 0
*CO1*	Hypocreales (order)	—	9.62	0.003	WW 278.25, 450.28; D 0.5, 0.87
FITS	Stachybotryaceae	*Stachybotrys* (genus)	24.2	<0.001	WW 279.5, 484.11; D 0, 0
18S	Ascomycota	—	6.96	0.011	WW 149.75, 141.39; D 0.75, 0.43
**Overrepresented in droughted (D)**					
16S	Burkholderiaceae	—	–4.06	0.048	WW 3, 2.35; D 25.5, 40.71
16S	Micropepsaceae	—	–1.85	<0.001	WW 115.75, 67.86; D 211.25, 99.91
16S	Oxalobacteraceae	*Massilia* (genus)	–4.43	0.019	WW 1.25, 1.30; D 14.0, 9.08

### Comparison of eDNA from greenhouse and field soil samples shows distinct communities

**Figure 3:**
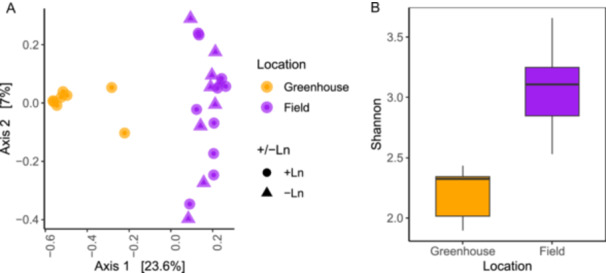
(A) Diversity of microbial communities in greenhouse and BLEA soil samples. (B) Beta diversity of fungal ITS sequences visualized using a Jaccard PCoA plot of greenhouse and field samples. Shannon's evenness index was higher for the field samples than for the greenhouse samples.

**Figure 4:**
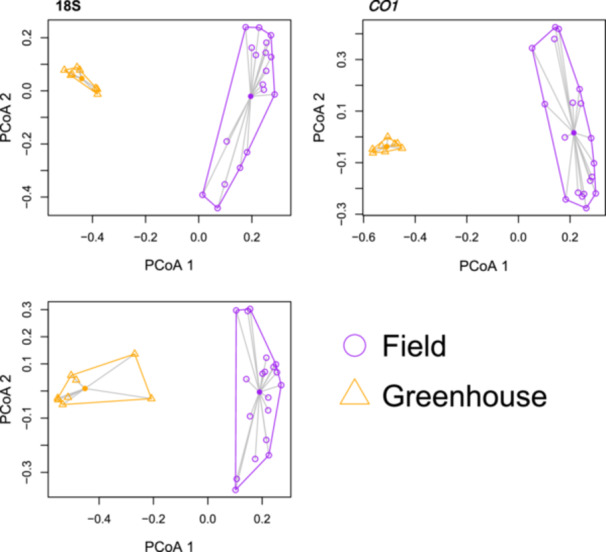
Beta dispersion analysis comparing greenhouse and Black Lake Ecological Area soil samples for fungal ITS, *CO1*, and 18S rRNA. Significant differences in beta dispersion are shown, indicating distinct clustering patterns between the two sample groups and suggesting pronounced dissimilarities in microbial community composition. These findings shed light on the ecological distinctiveness of the greenhouse and BLEA environments, emphasizing the importance of considering their unique microbial community structure.

Because we did not find any taxonomic alpha diversity between +Ln and −Ln samples or between microhabitat conditions, we grouped all field samples and compared the field group with grouped greenhouse plot samples with *L. nipomensis* growing in different microhabitat conditions. We found that species richness (Chao1) differed in communities detected using FITS and *CO1* between greenhouse and field samples. Evenness (Shannon) also varied in communities detected using FITS, *CO1*, and 16S but not 18S (Table 5). In both tests, field soils had greater Shannon and Chao1 values than greenhouse soil did. We found significant differences in the beta diversity of taxa between greenhouse and field samples (Table 6). For FITS, *CO1*, and 18S, beta dispersion was significantly greater in the field group versus the greenhouse (Figures 3, 4; Appendix S5).

**Table 5:** Results of *t*‐tests of alpha‐diversity statistics using the Chao1 and Shannon Indices comparing greenhouse to field samples. FITS, fungal ITS.

Index	Marker	df	*t*	*P*
Chao1	16S	21.530	0.325	0.748
Chao1	18S	22.790	1.507	0.146
Chao1	FITS	25.000	7.211	<0.001
Chao1	*CO1*	24.930	3.867	<0.001
Shannon	16S	21.390	–4.782	<0.001
Shannon	18S	23.270	1.021	0.318
Shannon	FITS	19.370	8.490	<0.001
Shannon	*CO1*	15.990	2.875	0.011

**Table 6:** PERMANOVA results testing beta‐diversity statistics using Jaccard dissimilarity indices comparing greenhouse to field samples.

Marker	df	*F*	*P*
16S	21.000	11.103	<0.001
18S	25.000	5.717	<0.001
FITS	25.000	6.277	<0.001
*CO1*	25.000	10.876	<0.001

## DISCUSSION

eDNA‐based biodiversity assessment of the soil biome is gaining traction for assessing ecological restoration, but thus far, work has hardly focused on endangered plants (van der Heyde et al., 2022). This study is among the initial studies to apply eDNA metabarcoding of the soil biome to inform endangered plant restoration efforts and to compare plant–microbe–animal relationships in situ and ex situ (Johnson et al., 2023). We highlight the potential for eDNA to be a tool in conservation and restoration efforts that focus on a particular species. Our results suggest that microhabitats can play an important role in influencing taxonomic beta diversity of soil organisms, as seen with the *CO1* and FITS markers (Table 1). This finding is consistent with previous research, which demonstrated that cool‐wet microhabitats consistently had higher species richness compared to warm‐dry areas (Luong et al., 2019; Lamprecht et al., 2021). However, whether the plots contained *L. nipomensis* did not have any discernible impact on the taxonomic alpha or beta diversity of the soil communities. This result suggests that there is no major difference in recruitment of the soil biome by *L. nipomensis*, meaning where it thrives may be a combined product of chance dispersal and preexisting conditions (Donath et al., 2003). Contrary to our initial expectations, we found an overrepresentation of nutrient‐cycling microbes but not nitrogen‐fixing microorganisms in the BLEA samples.

Greenhouse and field soil communities were distinct from each other, with field soil microbial communities being richer and more diverse. Initial use of phyloseq showed an overlap in microbial taxa between the greenhouse and field environments, suggesting both share a similar microbial community despite their different settings. The overlap in microbial taxa implies that the same microbes can be found in both locations, likely due to core microbes unique to the plant (Berg and Smalla, 2009). However, when we applied DESeq2 to examine how our treatments affected microbial abundance within each environment, none of the significantly upregulated or downregulated taxa overlapped between these conditions, which suggests that soil‐biome moisture levels can be influenced by environmental conditions. The lack of shared differentially abundant taxa between the environments with the different moisture conditions points to the importance of local factors in determining how microbial communities adapt to moisture stress, emphasizing that microbial adaptation is closely tied to the specific environment they inhabit (Shade et al., 2012; Mandakovic et al., 2018).

### Microhabitats affect the alpha and beta diversity of microbes in soils of *Lupinus nipomensis*

We found differences in species composition when using *CO1* as a biomarker in eDNA samples from different microhabitats, representing taxonomic beta diversity. Past work on soil fungal communities in nitrogen‐enriched soil showed that plant diversity was coupled with the taxonomic beta diversity of soil fungal microbes (Prober et al., 2015). The diversity of plant species within *L. nipomensis* microhabitats may mirror variation in soil fungal diversity. In other words, environmental conditions and surrounding plant communities might influence the taxonomic diversity of the biome in *L. nipomensis* soils, and future studies should investigate this possibility. Furthermore, we saw marginal differences in taxonomic beta diversity for the fungal taxa in *L. nipomensis* soils between wet‐cool and dry‐warm plots. A diverse array of soil communities allows ecosystems to be more resilient and better able to adjust to changes in environmental conditions (De Vries and Shade, 2013). For sugarcane (*Saccharum* spp.), the diversity of nitrogen‐fixing rhizobacteria was associated with growth enhancement (Singh et al., 2020). While most studies to date were conducted in agricultural settings, we observed greater diversity in fungal communities in soils of *L. nipomensis* when using the FITS marker compared to other markers. This finding indicates that different plant species and their environments can influence the composition and diversity of beneficial fungal communities, potentially leading to different impacts on plant health and growth.

### Microbial community structure in relation to microhabitats and + Ln/−Ln

We found that the microbes associated with *L. nipomensis* will vary in overrepresentation based on temperature, moisture, and +Ln/−Ln. Certain microbial groups were more abundant in specific microhabitats and in plots with either +Ln or −Ln. We did not find any similarities in the overrepresentation of microbial groups between different microhabitats or between +Ln and −Ln plots. This absence of distinct microbial communities, especially as a non‐carpeting form of Fabaceae (i.e., unlike soybeans), shows the interplay between plant species and their surrounding environment. The challenging conditions—predominantly sandy soils and barren landscapes, combined with drought—seem to limit its nitrogen‐fixation capabilities (Bordeleau and Prévost, 1994; Winterhalder, 1996).

The overrepresented microbes we found in dry‐warm field microhabitats within the family Pseudonocardiaceae might play a role in regulating soil carbon cycle processes (Appendix S6), and further studies are needed to confirm the specific functions of these microbes. The recruitment of these microbes could be a response to the dry‐warm conditions because climate stressors influence plant–soil feedback by altering plant inputs such as litter and root exudates and the composition of living plant roots and their mutualistic symbionts (Pugnaire et al., 2019). In wet‐cool microhabitats, we detected microbes belonging to the class Gammaproteobacteria, which includes members known to stimulate plants to produce salicylic acid, a hormone essential for plant defense, and to assist in sulfur oxidation and nitrate/nitrite reduction (Appendix S6). However, given the diversity within this class, further studies are needed to confirm the specific functions of these microbes in our samples. Plant pathogens often thrive in environments with high precipitation, air humidity, and soil moisture, which can increase the pathogenic potential of microorganisms that infect aboveground plant tissues (Velásquez et al., 2018). In our wet‐cool eDNA samples, we detected plant pathogens and immunological‐response microbes, but further investigation is needed to determine the species and their roles.

Geodermatophilaceae, specifically *Modestobacter* spp., play a role in carbon and nutrient cycling and are found in response to temperature stress (Appendix S6). Bogati and Walczak (2022) found that drought can decrease enzymatic activity of soil microbes, nutrient cycling, and soil fertility, all of which reduce plant productivity. In our +Ln plots, we found *Cladorrhinum australe* (Lasiosphaeriaceae), which may play a role in controlling phytopathogens and promoting plant growth, suggesting that some microbes might still contribute to these processes despite drought conditions. Microorganisms have been shown to reduce the impact of environmental stress on plants by directly supplying essential nutrients (Goh et al., 2013). Furthermore, these microorganisms can indirectly influence plant development and defense responses, promoting plant fitness and phenotypic plasticity (Goh et al., 2013). Our findings show that soil microbial overrepresentation differs between +Ln and −Ln plots. Expanded sampling and analysis might detect more microorganisms that contribute to the success of *L. nipomensis*.

### Lack of overlap in soil microbial communities between in situ and greenhouse soils

Overall, we observed an overrepresentation of nutrient‐cycling microbes in dry‐warm treatments at BLEA but not of nitrogen‐fixing bacteria as we originally hypothesized. While our Pro‐Mix HP Agtiv Reach potting soil is noted to contain *Rhizophagus irregularis* (formerly *Glomus intraradices*), this fungus was not overrepresented in our DESeq2 analysis. Nitrogen‐fixing bacteria are temperature‐sensitive, and high temperatures can stifle microbial activity (Kantar et al., 2010). Since nitrogen‐fixing bacteria need moisture to multiply and operate, changes in soil moisture levels may have an adverse effect on population sizes (Fierer and Schimel, 2002). Nitrogen‐fixing bacteria may experience competition for essential nutrients and space from one or a combination of other microbes, weeds, and nitrogen‐fixing plants (Kantar et al., 2010). The competition between microbes and plants is centered around the limited availability of resources, including photosynthate, phosphorus, and nitrogen, which are crucial for both microbial growth and plant development (Bauer et al., 2012). Unlike in the field, in our greenhouse drought treatments,we saw nitrogen‐fixing microbes from *Massilia* (genus), which can promote plant growth and nitrogen acquisition. Furthermore, in our greenhouse well‐watered treatments, we found microbes from the order Nitrosomonadales, an order that contains many nitrogen‐fixing bacteria species (Appendix S6).

Restoration projects often employ greenhouses for the *ex situ* propagation of rare or endangered plant species in controlled environments (Gibson et al., 1999). The ultimate objective is to nurture these plants to a viable size for subsequent introduction into their natural habitat or to produce seeds for future *in situ* efforts. However, there are differences in plant–soil interactions between greenhouse and field (Forero et al., 2019), potentially resulting in the underperformance of reintroduced plants due to microbial mismatch (Rauschkolb et al., 2019). This discrepancy can lead to the failed establishment and survival of the reintroduced plants in their new environment.

While also subjected to water and drought stress, our results showed *L. nipomensis* had nitrogen‐fixing microbes present in our greenhouse soils and overall had no overlap with the BLEA samples. The conditions within a greenhouse are frequently more favorable for microbes such as nitrogen‐fixing bacteria (Forero et al., 2019). The rapid differentiation between wild and *ex situ* origins and the effects of adaptation to cultivation conditions can lead to a mismatch between greenhouse‐grown plants and field plants (Rauschkolb et al., 2019). We acknowledge that nutrient‐cycling microbes may supply N from other sources because BLEA is about 2490 m from the ocean and 700 m from the nearest road. Fabaceae plants inherently form symbiotic relationships with nitrogen‐fixing bacteria; thus, the complex interactions within soil and root nodules suggest a nuanced continuum of microbial support that may play a crucial role beyond traditional symbiosis, especially in nutrient‐poor environments like those observed at BLEA. These interactions suggest that, despite their ability to fix nitrogen, these plants may still benefit from additional nitrogen sources facilitated by diverse microbial communities (Van Der Heijden et al., 2008). The greenhouse study showed lower species richness and evenness in comparison to the BLEA samples, highlighting the issue that *ex situ* plants may have been grown in soil that lacks the microbes necessary for optimal growth and survival in the wild.

### Future work and limitations

Despite this progress, our understanding of the functions of soil biodiversity is still in its early stages. We recognize that our study involved a limited sample size, which presents an opportunity for future research to explore these findings through expanded sampling across multiple time periods. Furthermore, to improve the accuracy of the results, we need to optimize sample processing to prevent the loss of DNA, and updated reference databases for microbes will reduce the number of “unknown” microbes during bioinformatics (Pawlowski et al., 2020). Additionally, the eDNA bioinformatic tools employed are currently not user‐friendly and need to be improved to make biological monitoring more readily and easily accessible (Pawlowski et al., 2020). Future work on soil biomes may benefit from analysis on platforms that make results easy to generate and readily comparable across studies, such as the new eDNAExplorer.org, but the website was not available at the time this work was done.

## CONCLUSIONS

Our results show that microhabitats may influence soil taxonomic beta diversity and dispersion but not alpha diversity of soil microbes. Certain taxa were overrepresented across microhabitats and between +Ln and −Ln plots, suggesting that the conditions in these microhabitats and legacy effects might influence the overrepresentation of specific taxa. However, whether plots were +Ln or −Ln was not reflected in the taxonomic alpha and beta diversity of the soil microbial communities. Focusing on these statistics alone would have missed interesting plant‐soil taxa relationships.

Conservation programs often use greenhouses to cultivate endangered plant species in controlled environments, aiming to reintroduce them into their native habitats. However, our study suggests that restoration efforts could be enhanced by considering the differences in soil microbial communities between the greenhouse and field soils. These differences, coupled with the influence of adaptation to cultivation conditions, could potentially reduce the survival of greenhouse‐grown plants when introduced to field conditions. We need to further explore the role of soil communities in plant growth and survival when reintroducing plants from greenhouses to their natural habitats.

## AUTHOR CONTRIBUTIONS

P.T.N. and J.C.L. conceived the greenhouse and experimental design; J.C.L. and L.C.S. conceived the field design, P.T.N., J.C.L., R.S.M., and M.E.L. conceived the research ideas; P.T.N., J.C.L., and V.W. collected data, P.T.N., R.S.M, and J.C.L. analyzed the data; P.T.N. led the writing of the manuscript with editorial contributions from J.C.L., M.E.L. and R.S.M. All authors contributed critically to the drafts and gave final approval for publication.

## Supporting information


**Appendix S1.** Map of Black Lake Ecological Area with sampling locations. Samples were taken from 20 different plots in either dry‐warm or wet‐cool microhabitats and +/−Ln plots.

**Appendix S2.** Black Lake Ecological Area plot sampling. In each plot, three subsamples were taken at least 5 cm apart and at a depth of 5 cm.

**Appendix S3**. Metabarcoding markers and primers used in this study.

**Appendix S4.** Metabarcoding results for 16S, 18S, *CO1*, and fungal ITS and sample metadata for field location and sample type. After sequencing, we assigned taxa using the Anacapa toolkit to construct detailed reference databases and taxonomically classify multilocus metabarcode sequence data. Samples were decontaminated, and taxa were filtered to only be retained if they were present in at least two samples and had at least 20 reads in the full data set per marker. During analysis, we filtered these further to remove samples with fewer than 1000 reads.

**Appendix S5.** Beta dispersion test for 16S, 18S, *CO1*, and fungal ITS, with 99 permutations in the permutest to look for differences in beta dispersion between variables. This test evaluates the homogeneity of group variance in metabarcoding‐based community composition to determine whether there are significant differences in variance. Beta dispersion does not rely on specific distributional assumptions.

**Appendix S6.** Taxa identified using DESeq2, which analyzes microbial taxa abundance in different conditions (e.g., wet vs. dry) using a negative binomial model to handle count variability and sampling depth differences. It detects significant taxa abundance shifts through a Wald test, focusing on adjusted log_2_ FoldChanges. This test was done for all four makers. A positive log_2_ FoldChange indicated wet plants, while a negative value indicated dry plants. The last column indicates the lower classification and known functions of the microbes. Known functions for taxa are included if we found them in literature review.

## Data Availability

All sequence data from this study are deposited in NCBI under project PRJNA1218458 (https://www.ncbi.nlm.nih.gov/sra/PRJNA1218458).
