# Supplementary material for: Soil biome variation of Lupinus nipomensis in wet‐cool vs. dry‐warm microhabitats and greenhouse
Source: Am J Bot. 2025 Mar 21;112(4):e70020. doi: 10.1002/ajb2.70020 (PMC12012791; doi:10.1002/ajb2.70020)
Supplement: Supplementary file 1 — Appendix S1. Map of Black Lake Ecological Area with sampling locations. Samples were taken from 20 different plots in either dry‐warm or wet‐cool microhabitats and +/−Ln plots. [file AJB2-112-e70020-s002.pdf]

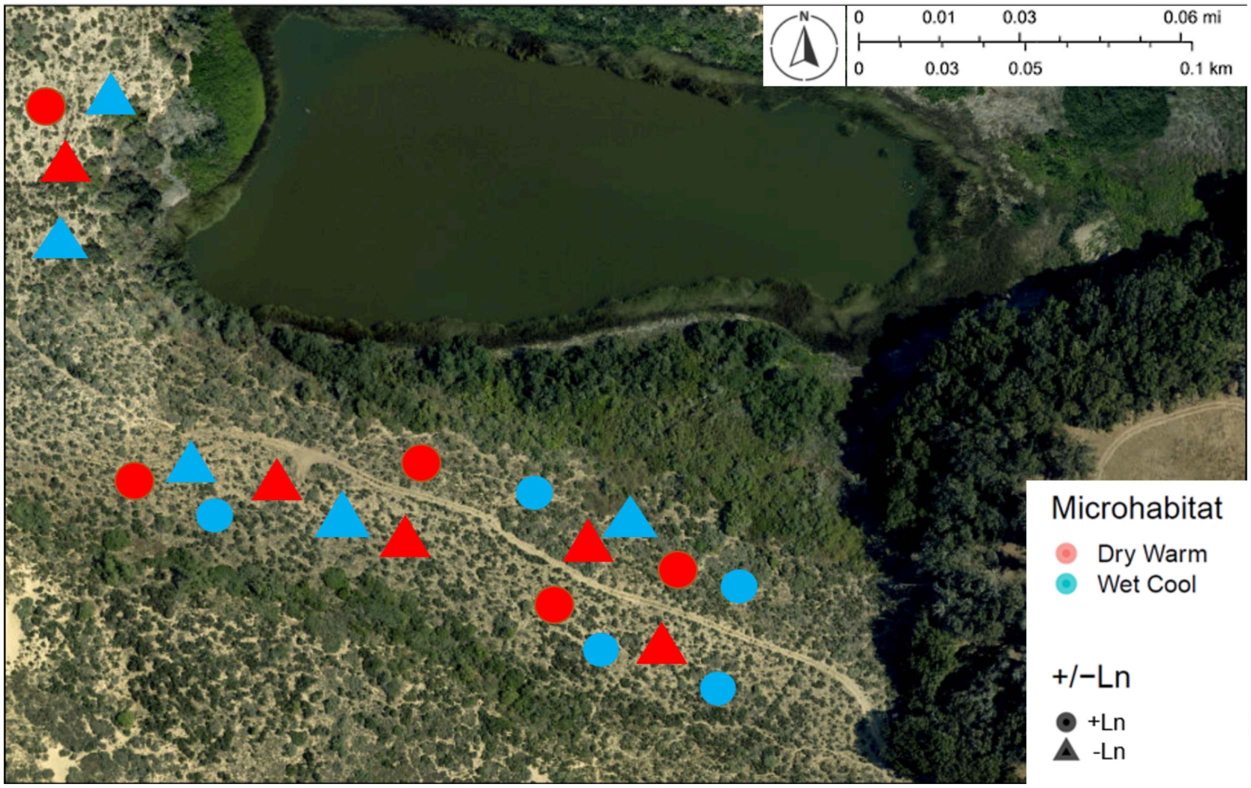

Appendix S1. Map of Black Lake Ecological Area with sampling locations. Samples were taken from 20 different plots in either dry-warm or wet-cool microhabitats and +/-Ln plots.
