# Supplementary material for: Soil biome variation of Lupinus nipomensis in wet‐cool vs. dry‐warm microhabitats and greenhouse
Source: Am J Bot. 2025 Mar 21;112(4):e70020. doi: 10.1002/ajb2.70020 (PMC12012791; doi:10.1002/ajb2.70020)
Supplement: Supplementary file 2 — Appendix S2. Black Lake Ecological Area plot sampling. In each plot, three subsamples were taken at least 5 cm apart and at a depth of 5 cm. [file AJB2-112-e70020-s005.pdf]

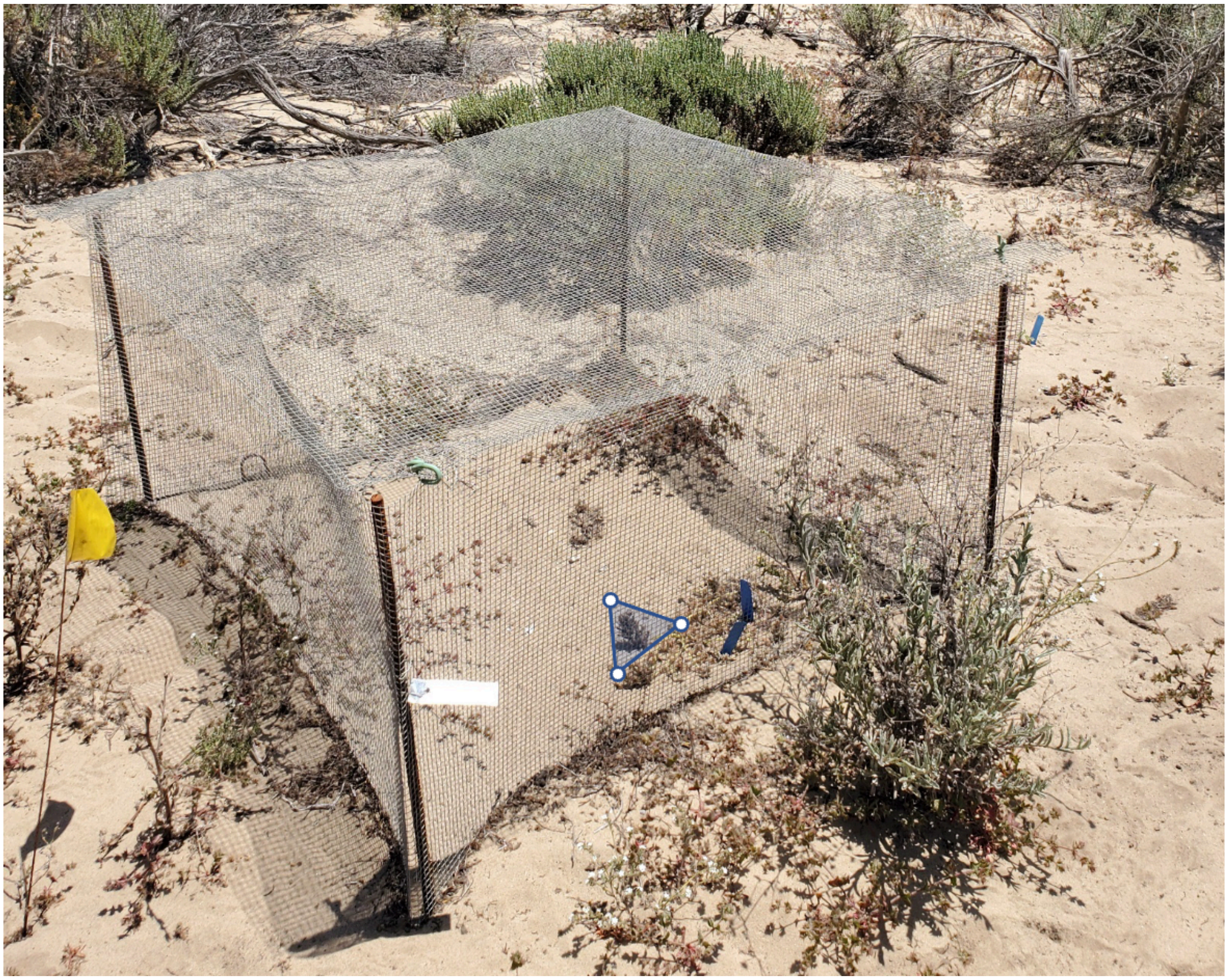

Appendix S2. Black Lake Ecological Area plot sampling. In each plot, three subsamples were taken at least 5 cm apart and at a depth of 5 cm.
